# Vitamin D and Calcium Status in South African Adolescents with Alcohol Use Disorders

**DOI:** 10.3390/nu4081076

**Published:** 2012-08-20

**Authors:** Celeste E. Naude, Paul D. Carey, Ria Laubscher, George Fein, Marjanne Senekal

**Affiliations:** 1 Division of Human Nutrition, Faculty of Medicine and Health Sciences, Stellenbosch University, Tygerberg Campus, Western Cape Province 7500, South Africa; 2 Department of Psychiatry, Faculty of Medicine and Health Sciences, Stellenbosch University, Tygerberg Campus, Western Cape Province 7500, South Africa; Email: vcarey18@telus.net; 3 Biostatistics Unit, South African Medical Research Council, Tygerberg, Western Cape Province 7500, South Africa; Email: Ria.Laubscher@mrc.ac.za; 4 Neurobehavioral Research Inc., 1585 Kapiolani Blvd, Honolulu, HI 96814, USA; Email: george@nbresearch.com; 5 Division of Human Nutrition, Department of Human Biology, University of Cape Town, Observatory, Western Cape Province 7935, South Africa; Email: Marjanne.Senekal@uct.ac.za

**Keywords:** vitamin D, calcium, adolescent, alcohol, skeletal health

## Abstract

Adequate vitamin D and calcium are essential for optimal adolescent skeletal development. Adolescent vitamin D insufficiency/deficiency and poor calcium intake have been reported worldwide. Heavy alcohol use impacts negatively on skeletal health, which is concerning since heavy adolescent drinking is a rising public health problem. This study aimed to examine biochemical vitamin D status and dietary intakes of calcium and vitamin D in 12–16 year-old adolescents with alcohol use disorders (AUD), but without co-morbid substance use disorders, compared to adolescents without AUD. Substance use, serum 25-hydroxyvitamin D (s-25(OH)D) concentrations, energy, calcium and vitamin D intakes were assessed in heavy drinkers (meeting DSM-IV criteria for AUD) (*n* = 81) and in light/non-drinkers without AUD (non-AUD) (*n* = 81), matched for age, gender, language, socio-economic status and education. Lifetime alcohol dose was orders of magnitude higher in AUD adolescents compared to non-AUD adolescents. AUD adolescents had a binge drinking pattern and “weekends-only” style of alcohol consumption. Significantly lower (*p* = 0.038) s-25(OH)D (adjusted for gender, smoking, vitamin D intake) were evident in AUD adolescents compared to non-AUD adolescents. High levels of vitamin D insufficiency/deficiency (s-25(OH)D < 29.9 ng/mL) were prevalent in both groups, but was significantly higher (*p* = 0.013) in the AUD group (90%) compared to the non-AUD group (70%). All participants were at risk of inadequate calcium and vitamin D intakes (Estimated Average Requirement cut-point method). Both groups were at risk of inadequate calcium intake and had poor biochemical vitamin D status, with binge drinking potentially increasing the risk of the latter. This may have negative implications for peak bone mass accrual and future osteoporosis risk, particularly with protracted binge drinking.

## 1. Introduction

Adolescents are recognised as a nutritionally at-risk group [1]. Specific nutritional vulnerabilities pertinent to adolescence include vitamin D and calcium status within the context of rapid skeletal development and the attainment of peak bone mass [2,3,4]. Calcium is the dominant mineral in bone, accounting for approximately 40% of bone mineral content [5]. The active form of vitamin D [1,25-dihydroxyvitamin D (1,25(OH)2D)] is essential for intestinal calcium absorption to meet the needs of skeletal growth and bone development [6,7]. Adequate concentrations of vitamin D results in intestinal dietary calcium absorption of about 30 to 40% [8,9], while the absence of vitamin D, results in absorption of only approximately 10 to 15% of dietary calcium [9]. The efficiency of renal calcium reabsorption is also increased in the presence of 1,25(OH)_2_D [9,10]. Furthermore, a decrease in serum 25-hydroxyvitamin D (s-25(OH)D) concentrations induces parathyroid hormone release, which increases osteoclast activity and bone resorption [9,11,12,13]. 

Adolescents appear to be at risk of vitamin D insufficiency and deficiency [2,14] and a high prevalence of inadequate vitamin D status in older children and adolescents continues to be reported globally in developed and developing countries [15,16,17,18,19]. Severe and prolonged clinical vitamin D deficiency results in rickets in children and osteomalacia in adults [20]. It must be borne in mind that accrual of more than 90% of peak bone mass occurs during adolescence [12], emphasizing the need for adequate calcium intake during this life stage [4,21]. Peak bone mass is a significant determinant of risk for fractures and osteoporosis in later life [3,22]. Adolescents with insufficient vitamin D and calcium nutriture and the associated increased risk for low bone mass, are at an increased risk for the development of osteoporosis later in life [4,23]. 

Alcohol *per se* is also known to negatively affect bone health. Inhibition of bone growth with heavy alcohol intake has been observed in experimental animals [24] and also in humans, in whom a shorter stature was seen in those who drank during growth (birth to 18 years of age) compared to those who did not [25]. Alcohol abuse disturbs osteoblastic activity and the decrease in bone mass and strength following heavy alcohol intake is primarily due to a bone remodeling imbalance, with a predominant reduction in bone formation [26,27,28]. 

In South Africa, the legal age limit for alcohol use is 18 years, but surveillance reveals heavy alcohol use by adolescents, which represents a significant public health concern [29,30,31,32]. The most recent South African Youth Risk Behaviour Survey (YRBS) reported that half (49.6%) of the national sample of grade 8 to 11 adolescents (*n* = 10,270) had used alcohol and that this prevalence increased with age, with 35% of adolescents reporting current use of alcohol. In terms of region, the Western Cape Province had the highest prevalence of current drinkers (53%) [31]. Episodic consumption of large quantities of alcohol, generally termed binge drinking, is the common drinking pattern among adolescents and usually includes multiple binge drinking episodes, typically in a “weekends-only” drinking style [29,30,31]. Nationally, 29% of adolescents in the 2008 South African YRBS reported binge drinking in the month preceding the YRBS [31], which is a significant increase from the rate of 23% in the 2002 YRBS [32]. In all likelihood, persistent heavy binge drinking by adolescents is likely to be a significant predictor of health outcomes, including those related to nutritional health. 

Currently little is known about the association between heavy alcohol use during adolescence, specifically in the form of binge drinking, and vitamin D and calcium nutriture. Furthermore, there is a paucity of information on vitamin D and calcium nutriture in adolescents in South Africa, particularly in the more southern latitudes of the country. The aim of this study therefore was to examine the biochemical vitamin D status, as well as the dietary intake of calcium and vitamin D of treatment-naive (no interventions for alcohol use), 12 to 16 year-old community-based adolescents with alcohol use disorders (AUD), but without co-morbid substance use and psychiatric disorders (SUD), in comparison to light/non-drinking adolescents without AUD, from the same well-defined and homogenous study population. The inclusion of adolescents without co-morbid externalizing disorders or SUD allowed the study of the relationships between AUD and vitamin D and calcium indicators without the confounding effects of other SUD or externalizing disorder risk factors. 

## 2. Methods and Materials

### 2.1. Study Population and Participants

The sample (*n* = 162) consisted of low socio-economic status English or Afrikaans-speaking adolescents (ages 12 to 16 years) from schools within a 25 km radius of Tygerberg Hospital located in the greater metropolitan area of Cape Town, Western Cape, South Africa. Screening included a structured psychiatric diagnostic interview, a developmental and medical history (from participants and at least one biological parent or legal guardian), a detailed physical and neurological examination assessing developmental delays and urine analysis and breathalyser testing (to confirm sobriety of participants during testing procedures). The Schedule for Affective Disorders and Schizophrenia for School Aged Children (6 to 18 years) Lifetime Version (K-SADS-PL) [33] was used to screen for psychiatric diagnoses. The Semi-Structured Assessment for the Genetics of Alcohol (SSAGA-II) [34] was used to confirm AUD diagnosis and to derive detailed substance use histories (alcohol, tobacco and all other drugs).

Participants were recruited for an AUD group meeting DSM-IV criteria for alcohol dependence or alcohol abuse [35] and for a light/non-drinking non-AUD group (with lifetime dose of <100 standard drinks of alcohol). Exclusion criteria for both groups were: mental retardation, lifetime DSM-IV diagnoses other than AUD, as defined in the KSADS-PL, current use of sedative or psychotropic medication, current signs or history of foetal alcohol spectrum disorders or exposure to heavy antenatal alcohol, sensory impairment, history of traumatic brain injury with loss of consciousness exceeding 10 min, presence of diseases that may affect the CNS (e.g., meningitis, epilepsy), HIV (tested using the enzyme linked immunosorbent assay (ELISA)), less than 6 years of formal education, and lack of proficiency in English or Afrikaans. Prior to consent being obtained for participation in the study, a social worker obtained collateral information from consenting parents, verifying the absence of medical, psychiatric and psychosocial problems. Participants in the two groups were individually matched for age (within 1 year), gender, language, socio-economic status and level of education (within 1 year). A total socio-economic status score was calculated for each participant by summing the category scores for family income (1–6), reversed employment category of participant's parent with the highest employment rank (Hollingshead reversed) (1–9), parent education (0–6), total assets (0–7), dwelling type (1–6) and bedroom cohabitation (1–7), with a maximum score of 41. Scores increased as indicators of socio-economic status increased, thus a higher score was associated with a higher SES. During recruitment it was attempted to match the samples for smoking status, but this was not feasible since smoking was much more prevalent in the AUD participants. This positive association of smoking and alcohol use is well documented [36]. 

### 2.2. Measures

***Substance Use***: A revised version of the Timeline Follow-back procedure (TLFB) [37], a semi-structured, psychiatrist-administered assessment of lifetime history of alcohol use and drinking patterns (*i.e*., frequency, quantity and density of alcohol consumption, including every phase from when participants first started drinking at least once per month to the present, including all periods of abstinence) was used in combination with the K-SADS-PL to elicit alcohol-use data. A standard drink was defined as one beer, cider or wine cooler (340 mL), one glass of wine (150 m) or a 45 mL shot of liquor. A similar procedure was carried out for each substance that the research participant acknowledged using.

***Biochemical Determination of Vitamin D Status***: Circulating s-25(OH)D is widely regarded as the most appropriate measure of vitamin D status with concentrations reflecting medium to long term vitamin D availability from both endogenous and dietary sources [11]. EDTA venous blood samples for the s-25(OH)D assays were collected from each consenting participant via venipuncture. Recruitment of subjects took place across all four seasons of the year resulting in a spread of blood sampling for assessment of vitamin D status over all the seasons. The LIAISON 25(OH) vitamin D assay (DiaSorin, Stillwater, MN, USA) was performed on the LIAISON analyser according to the manufacturer’s instructions. The analytical runs for control and sample specimens were done in duplicate. 

For the interpretation of s-25(OH)D concentrations, it is important to note that there is no consensus in the literature on concentrations of s-25(OH)D that define vitamin D insufficiency in infants and children [38]. Holick and Chen have assumed that children have similar requirements to adults [39]. In line with this assumption, a s-25(OH)D concentration of greater or equal to 20 ng/mL was used in establishing the Institute of Medicine (IOM) Recommend Dietary Allowance for vitamin D to meet the requirements of nearly all children and adolescents [20,38,40]. The cut-offs for s-25(OH)D concentrations used in adults and in this study to define vitamin D status, have been used to classify vitamin D status in children [41]. These categories are based on data showing that intestinal calcium absorption is maximised above 32 ng/mL [8] and that parathyroid hormone concentrations in adults continue to decline and reach their nadir at between 30 and 40 ng/mL [42,43,44]. The following categories were thus used to define vitamin D status based on s-25(OH)D concentrations: vitamin D deficiency (<20 ng/mL), insufficiency (20 to 29.9 ng/mL) and sufficiency (≥30 ng/mL and greater) [9,45,46]. These categories are also in line with the reference ranges recommended for the LIAISON 25 OH Vitamin D assay.

***Energy, Calcium and Vitamin D Intake***: Energy, calcium and vitamin D intakes were estimated using three 24-h recall questionnaires per participant. This method of dietary intake assessment has been shown to be appropriate for quantifying dietary intake of groups in developing countries [47,48] and internal and external validity has been found to be acceptable in adolescents aged ten years and older [49,50].

The energy (kilojoules (kJ)), calcium (milligrams (mg) and vitamin D (International Units (IU)) intakes for each participant for each day were calculated using the South African Food Data System (SAFOODS) [51]. The average intakes over the three 24-h recall interviews were calculated to represent the observed intake distributions. The dietary data include nutrient intake estimates from food (both naturally present and fortified) and water only and exclude nutrient intake estimates contributed by dietary supplements and medications or that obtained from sunlight. It is important to bear in mind the limitations of the vitamin D intake estimates in this study, namely the relatively high percentage of missing values for vitamin D (approximately 30% to 40%) in the SAFOODS database [52].

The energy intake variable obtained from the 24-h recalls did not include estimated energy from alcohol intake. Average daily alcohol energy intake of the AUD group was estimated from average daily alcohol intake (g) per participant in the AUD group using the alcohol-use data from the most recent phase of drinking as follows: (1) frequency of alcohol use (days per month) multiplied by average quantity of alcohol consumed (standard drinks per drinking day) to obtain average monthly standard drinks of alcohol consumed; (2) average monthly standard drinks of alcohol consumed was divided by 28 days to obtain average daily standard drinks of alcohol consumed; (3) average daily standard drinks of alcohol consumed was multiplied by 13.6 g of alcohol per standard drink to obtain average daily alcohol intake in grams, which was converted to average daily alcohol energy (29 kJ/g) to obtain average daily alcohol energy (kJ). Average daily alcohol energy was added to daily energy intake from the observed intake distributions for each AUD participant to represent total estimated energy intake. Daily alcohol energy for the *n* = 48 light drinking participants in the non-AUD group was not calculated as their alcohol life dose was negligible (mean 5.77; SD 12.46 standard drinks), and the contribution of alcohol energy to total estimated energy intake would therefore also be negligible. The total estimated energy intake variable was reported in this study only for use when regression-adjusted differences between groups were determined, since differences in energy intake between groups may confound differences in nutrient intakes. The 24-h recall data was further used to determine the top five foods foods/energy-containing beverages (excluding alcoholic beverages) that contributed to calcium intake in each group. 

***Eating Behaviour (Frequency of Intake of Indicator Foods)***: The weekly frequencies of intake of foods reflecting healthy and poor food choices were estimated using a non-quantitative food frequency questionnaire. The questionnaire consisted of a list of 37 food categories, with each food category consisting of either single or multiple food items that were grouped based on shared nutritional characteristics. Indicator foods/categories were identified by firstly listing foods most commonly consumed by South Africans in the Western Cape using scientific reports, publications as well as unpublished dietary assessment information generated in small research projects/compilation of community profiles for nutrition interventions. Identified foods/categories were then classified as either healthy choices (offer protective effects against non-communicable diseases (NCDs)) or as poor choices (would increase NCDs risk). A panel of nutrition and NCD health experts advised this process. The response categories included “eaten in the past month” (yes/no), and if yes, “times eaten per week” or “times eaten per month”.

The frequency of intake recorded for each one of the 37 food categories was converted to reflect the number of times eaten per week per participant. The food categories “milk/sour milk/yoghurt” and “cheese” were selected as indicators of calcium intake for the purposes of this paper, and weekly frequencies of intake for only these two food categories are reported.

### 2.3. Procedures

Recruitment procedures included oral presentations at schools and advertisement via word-of-mouth. A convenience sample of alcohol users was selected and non-users and light users for each of these adolescents were selected individually according to matching criteria. At the pre-screening stage, adolescents who did not meet eligibility criteria for possible inclusion in the AUD or non-AUD groups were excluded. Participants who met eligibility criteria were transported from their homes or schools to the testing site for complete physical and psychiatric screening for possible allocation to one of the groups or exclusion. 

After confirmation of inclusion in the study, demographic information was obtained, the first 24-h recall interview was conducted and blood samples for the biochemical determination of s-25(OH)D were obtained in the morning from each participant by a phlebotomist. The remaining two 24-h recall interviews were completed on a Monday to obtain Sunday intakes and one other week day thereafter. The 24-h recall interviews were all conducted by a trained researcher, versed in relevant terminology and locally available food and beverages. The procedure for the 24-h recalls included the following consecutive steps: (a) listing of foods and beverages (including water) consumed by the participant in the previous 24 h, starting from time of waking and proceeding chronologically until time of going to sleep; (b) collection of detailed description of foods, preparation methods and brands where relevant and the amounts consumed; and (c) final checking to recall forgotten foods. Commonly used household measures and food pictures from the Dietary Assessment and Education Kit, developed by Steyn and Senekal [53] were used to assist with food portion size estimation. Estimated food portions were converted to grams using the MRC Food Quantities Manual [54]. The 24-h recall interviews in this study were conducted over a period that included all seasons of the year to account for seasonal dietary variations. Dietary data could not be collected for Fridays and Saturdays as it was not feasible to conduct interviews on Saturdays and Sundays.

The indicator food frequency questionnaire was administered by the trained researcher during the second contact session that also involved administration of the second 24-h recall interview. 

### 2.4. Ethics

The Committee for Human Research of Stellenbosch University approved all study procedures (N06/07/128). After eligibility was established, written consent from parents and written assent from participants was obtained. Participants were compensated for their time with gift vouchers. Confidentiality of all study information was maintained with the exception of statutory reporting requirements in newly-identified or ongoing threats to the safety of minor participants. 

### 2.5. Statistical Analysis

Descriptive statistics, including inspection of data for adherence to normal distributions, and group comparisons were computed using Stata/IC Version 11.1 for Windows [55]. Suitable transformations were applied to all variables with skewed distributions. Statistical significance was defined at a level of *p* ≤ 0.05. The smoking variable used in all regressions included the smoking group (light smokers (lifetime < 100 cigarettes) and regular smokers (lifetime > 100 cigarettes)) and the non-smoking group (participants who have never used tobacco). 

Descriptive statistics (frequency distributions/means and standard deviations) of socio-demographic and substance use variables in the non-AUD and AUD groups were computed and compared for confirmation of group allocation and matching. For comparison of continuous variables, the Mann Whitney U Test was used and for categorical variables the Chi-square or Fisher’s exact tests were used. 

Descriptive analyses for s-25(OH)D concentrations, dietary intakes of energy, calcium and vitamin D as well as weekly frequency of intake of foods in the two food categories, namely “milk/sour milk/yoghurt” and “cheese” included medians and interquartile ranges for each group. Due to the paired nature of the data, multi-level mixed effects linear regression was used to compare these variables between the two groups, with adjustment for gender, smoking status and vitamin D intake. A pairing variable was created according to the matched pairs in the sample and served as the level variable in the model. The s-25(OH)D, energy, calcium and vitamin D variables (transformed) were used as the dependant variables in the regressions. The distribution of the weekly frequency of intake of “milk/sour milk/yoghurt” could not be suitably transformed and a rank variable was created for use as the dependant variable in this particular regression.

The frequencies of participants in the non-AUD and AUD groups with vitamin D sufficiency, insufficiency and deficiency (according to the defined vitamin D status categories) were computed and compared between groups, including gender groups, using the Chi-square test. 

As no specific reference intakes are available for the South African population, the Institute of Medicine’s (IOM) Dietary Reference Intakes (DRIs), were used to assess adequacy of dietary intake of calcium and vitamin D [20]. The adequacy of calcium and vitamin D intakes were determined using usual intake distributions in accordance with the recommendation of the IOM concerning the need to determine the usual nutrient intake distributions when assessing adequacy of dietary intake of groups in relation to the DRIs [56]. Adjustments to the observed intake distributions to obtain usual intake distribution estimates were made using the National Research Council (NRC)/IOM method [57,58].

Using the EAR cut-point method the frequencies of intakes below the EARs for calcium and vitamin D were computed in each group to reflect the risk of inadequate intake [56,59]. For these purposes, the EARs for 14 to 18 year-olds were used for calcium (1100 mg) and vitamin D (400 IU) [20]. The frequencies of usual intakes below the EARs (14 to 18 years) for each nutrient were compared between groups using the Chi-square test.

Using the 24-h recall data, the average calcium intake (milligrams) for each food code was computed for each participant, after which the average intake for each group was computed and ranked. This was done to obtain the top five foods/energy-containing beverages (excluding alcohol) that contributed to calcium intake in each group.

## 3. Results

### 3.1. Socio-Demographic and Substance Use Characteristics

A total of 184 adolescents were recruited and screened, of whom 22 were excluded as screen failures due to a range of exclusion criteria, including cannabis and methamphetamine use and DSM-IV Axis I diagnoses, resulting in a final sample of 162. The non-AUD and AUD groups were successfully matched for age, education level, gender, language and socio-economic status. All except two participants from the non-AUD group were from the mixed ancestry ethnic group (Table 1). 

**Table 1:** Confirmatory analyses of socio-demographic and alcohol grouping measures and substance use characteristics of the non-AUD and AUD groups.

	non-AUD(*n* = 81)	AUD (*n* = 81)	U/χ^2^	*p*-value
	*M* (*SD*) or %	*M* (*SD*) or %		
**Socio-demographics**				
Age	14.76 (0.78)	14.92 (0.74)	−1.19	0.235
Education level ^a^	7.79 (0.85)	7.85 (0.74)	−0.43	0.666
% Male	42	42	<0.01	1.000
% Female	58	58		
% Afrikaans-speaking	69	69	<0.01	1.000
% English-speaking	31	31		
% Mixed ancestry	97.6	97.6		0.497
% White	1.2	0		
% Black	1.2	0		
Total Socio-economic status score ^b^	28.19 (5.80)	24.85 (5.93)	1.34	0.179
**Alcohol Use**				
% Never consumed alcohol	41	0		
% Never intoxicated	93	0		
% Light drinker (Life dose < 100 standard drinks) ^c^	59	0		
% Alcohol abuse ^d^		2.5		
% Alcohol dependence ^e^		97.5		
% Weekends-only drinking style in most recent drinking phase ^f^		95%		
Drinking onset age (years) in participants that have drunk alcohol	12.25 (1.66)	12.04 (1.70)	0.57	0.567
Alcohol lifetime dose ^g^	5.77 (12.46)	1493.69 (1511.53)	−11.04	**<0.001**
Age of first intoxication		12.83 (1.15)		
Age of onset of regular drinking		12.91 (1.11)		
Regular drinking duration (months)		23.78 (15.91)		
Regular drinking frequency (days/month) in most recent drinking phase		5.01 (2.87)		
Regular drinking quantity/month (standard drinks) ^h^		65.78 (57.96)		
**Tobacco Use**				
% Never smoked tobacco	59	17		**<0.001**
% Light smokers (lifetime < 100 cigarettes)	35	31		
% Regular smokers (lifetime > 100 cigarettes)	6	52		
Smoking onset age (years) in light smokers	12.53 (1.62)	12.44 (1.96)	−0.19	0.846
Smoking onset age (years) in regular smokers	13 (0.71)	12.36 (1.46)	0.96	0.339
Lifetime tobacco dose of all smokers ^i^	86.42 (442.80)	1417.59 (2762.60)	−7.02	**<0.001**

**Abbreviation:** AUD: alcohol use disorders; **Notes:** For all variables not presented as percentages, means are presented with standard deviations in parentheses. Continuous variables compared using the Mann Whitney U Test and categorical variables compared using the Chi-square or Fisher’s exact tests. ^a^ Years of successfully completed education; ^b^ Total Socio-economic status score: Sum of Family income (1–6), Reversed employment category of participant’s parent with the highest employment rank (Hollingshead reversed) (1–9), Parent education (0–6), Total assets (0–7), Dwelling type (1–6) and Bedroom cohabitation (1–7)—Maximum = 41; ^c^ Less than 100 standard drinks of alcohol consumed in lifetime; ^d^ Greater than 100 standard drinks of alcohol consumed in lifetime with a DSM-IV diagnosis of alcohol abuse; ^e^ Greater than 100 standard drinks of alcohol consumed in lifetime with a DSM-IV diagnosis of alcohol dependence; ^f^ Style of drinking followed in the most recent phase of drinking; ^g^ Total number of standard drinks of alcohol consumed in lifetime; ^h^ Average standard drinks of alcohol consumed per month; ^i^ Total number of cigarettes smoked in lifetime.

As expected, AUD adolescents had dramatically greater alcohol exposure than those without AUD (Table 1). Almost all (95%) adolescents in the AUD group had a “weekends-only” style of alcohol consumption. The regular drinking frequency (days per month) and regular drinking quantity (standard drinks per month) indicates an approximate consumption of 13 drinks per drinking day, which suggests a binge drinking pattern. A greater proportion of adolescents in the AUD group smoked compared to the non-AUD group, and lifetime tobacco dose (total number of cigarettes smoked in lifetime) was greater in the AUD group (Table 1).

### 3.2. Biochemical Vitamin D Status

Blood samples for the biochemical determination of vitamin D status could be obtained from all participants in the sample, except for one AUD participant. Serum 25(OH)D was significantly lower in the AUD group compared to the non-AUD group (Table 2), although it is important to note that median s-25(OH)D concentrations in both groups were within the vitamin D insufficiency category (20 to 29 ng/mL) (Table 2). Vitamin D insufficiency and deficiency combined was found in almost 90% of AUD adolescents and approximately 70% of non-AUD adolescents (Table 2). A significantly greater percentage of adolescents in the AUD group had insufficient and deficient vitamin D status compared to the non-AUD group. There were no significant gender-by-group interaction effects for the frequency of adolescents with sufficient, insufficient and deficient vitamin D status for comparisons (Table 2). 

### 3.3. Vitamin D and Calcium Intake

Complete dietary intake data was collected for 160 participants, with two participants (one participant per group) refusing participation. Regressions showed significantly higher dietary intake of energy and calcium in the AUD group than in the non-AUD group, while vitamin D intake did not differ significantly between the two groups (Table 3). According to the EAR cut-point method all adolescents in the sample were at risk of inadequate calcium and vitamin D intakes, with intakes below the EARs for 14 to 18 year-olds (Table 4).

**Table 2:** Serum 25-hydroxyvitamin D concentrations and prevalence of vitamin D sufficiency, insufficiency and deficiency in the non-AUD and AUD groups, and comparisons between groups.

	Group		Males		Females	
	non-AUD	AUD	non-AUD	AUD	non-AUD	AUD
*Biochemistry*	**Median** ** (IQR)**					
**s-25(OH)D** (ng/mL)	25.7 *	**22.0** *	26.1	20.0	25.2	22.4
	(18.7–31.1)	(18.2–25.9)	(18.7–31.3)	(15.8–25.7)	(18.2–30.6)	(19.0–25.9)
*Categories of vitamin D status*	**Percentage *n***					
**Vitamin D sufficiency**	29.6 **	11.2 **	32.3	11.8	27.7	10.9
s-25(OH)D: ≥30 ng/mL	*24*	*9*	*11*	*4*	*13*	*5*
**Vitamin D insufficiency**	**28.4** **	**40.0** **	26.5	50.0	29.8	32.6
s-25(OH)D: 20 to 29.9 ng/mL	*23*	*32*	*9*	*17*	*14*	*15*
**Vitamin D deficiency**	**42.0** **	**48.8** **	41.2	38.2	42.5	56.5
s-25(OH)D: <20 ng/mL	*34*	*39*	*14*	*13*	*20*	*26*

**Abbreviations:** AUD: alcohol use disorders; IQR: interquartile range; s-25(OH)D: serum 25-hydroxyvitamin D; ng/mL: nanograms per millilitre; **Notes:** All variables had skewed distributions thus medians are reported with interquartile range (IQR) in parenthesis; *n*-values: non-AUD *n* = 81 (*n* = 34 males and *n* = 47 females); AUD *n* = 80 (*n* = 34 males and *n* = 46 females); * Significant differences between groups in s-25(OH)D (*p* = 0.038), using multilevel mixed-effects linear regression, adjusting for gender, smoking status and vitamin D intake; ** Significant differences between groups in frequencies of adolescents with sufficient, insufficient and deficient vitamin D status (*p* = 0.013), using Chi-square test.

**Table 3:** Estimated daily intakes of dietary energy, calcium and vitamin D (observed intake distributions) in the non-AUD and AUD groups, and comparisons between groups.

	Group		Males		Females	
	non-AUD	AUD	non-AUD	AUD	non-AUD	AUD
	Median (IQR)					
**Total Energy ^a^**	8965 *	**11** ,028 *	9461	11,684	8342	10,481
**(kJ)**	(7240–10,661)	(9072–13,014)	(8010–10,835)	(10,181–13,521)	(7003–9944)	(8847–11,726)
**Calcium **	450.1 *	460.0 *	525.4	508.9	438.1	415.9
**(mg)**	(347.8–614.1)	(334.1–627.1)	(391.1–651.4)	(442.8–722.0)	(318.4–564.8)	(330.7–534.1)
**Vitamin D **	99.2	120.1	123.3	134.9	83.6	112.9
**(IU)**	(64.6–160.1)	(83.6–193.6)	(95.2–180.5)	(86.5–216.3)	(60.3–131.3)	(83.1–163.4)

**Abbreviations:** AUD: alcohol use disorders; IQR: interquartile range; kJ: kilojoules; mg: milligrams; IU: International Units; **Notes:** All variables had skewed distributions thus medians are reported with interquartile range in parenthesis; *n*-values: *n* = 80 per group (males *n* = 33 and females *n* = 47); ^a^ Total estimated energy intake, including average daily alcohol energy estimated from average daily alcohol intake (grams) per participant in the AUD group; * Significant differences between groups in intakes of total energy (*p* < 0.001) and calcium (*p* = 0.007), using multilevel mixed-effects linear regression, adjusting for gender, smoking status and total estimated energy including alcohol (comparison of total energy between groups was only adjusted for gender and smoking status).

**Table 4:** Prevalence of risk of inadequate calcium and vitamin D intakes in the non-AUD and AUD groups using the EAR cut-point method, and comparisons between groups.

	EARs 14–18 years		non-AUD (*n* = 80)	AUD (*n* = 80)
	Males	Females	Prevalence below EAR (%)	Prevalence below EAR (%)
**Calcium** (mg)	1100	1100	100	100
**Vitamin D** (IU)	400	400	100	100

**Abbreviations:** AUD: alcohol use disorders; EAR: Estimated Average Requirement; mg: milligrams; IU: International Units; **Notes:** usual nutrient intake distributions used (statistically adjusted using National Research Council (NRC)/IOM method); No differences between groups in the numbers of adolescents with risk of inadequate calcium and vitamin D intakes, using Chi-square test.

The first four foods contributing most to calcium intake were the same in both groups and included whole milk, white bread, cheddar cheese, savoury maize and wheat crisps (Table 5). Frequencies of intake of “milk/sour milk/yoghurt” (times per week) in the non-AUD (median 7; IQR 7–7) and AUD (median 7; IQR 3–7) groups were not significantly different. There was also no difference in the frequencies of intake of “cheese” in the non-AUD group (median 2; IQR 1–3) compared to the AUD group (median 3; IQR 0.75–3).

**Table 5:** Top five foods/energy-containing beverages contributing to calcium intake (milligrams per day) in the non-AUD and AUD groups.

non-AUD Group (*n* = 80)			AUD Group (*n* = 80)		
Rank	Foods	Calcium (mg/day) ^a^	Rank		Calcium (mg/day) ^a^
1	Milk, full fat	143	1	Milk, full fat	121
2	Bread, white	50	2	Bread, white	63
3	Cheese, cheddar	47	3	Cheese, cheddar	50
4	Snack, savoury, wheat, maize crisps	21	4	Snack, savoury, wheat, maize crisps	27
5	Macaroni cheese	12	5	Custard (whole milk, custard powder)	19

**Abbreviation:** mg: milligrams; ^a^ Using the 24-h recall data, the average calcium intake (milligrams) for each food code was computed for each participant, after which the average intake for each group was computed and ranked.

## 4. Discussion

This study reports on the vitamin D and calcium status of a group of treatment-naive, 12 to 16 year-old community-based adolescents of mixed ancestry, with “pure” AUD, in comparison to a matched group of light/non-drinking adolescents. 

Serum 25(OH)D concentrations in the AUD adolescents were significantly lower than in non-AUD adolescents, although median serum concentrations were below the cut-off of 30 ng/mL in both groups. Furthermore, vitamin D insufficiency and deficiency, based on s-25(OH)D concentrations, were high in both groups, but significantly more so in the AUD group. Nearly half of AUD adolescents (48.8%) and 42% of non-AUD adolescents were classified as being vitamin D deficient (s-25(OH)D concentrations < 20 ng/mL). These findings suggest that both groups of adolescents of mixed ancestry in Cape Town have a poor vitamin D status, which may be exacerbated by heavy binge drinking alcohol use. 

The possibility that the heavy alcohol use by the AUD adolescents may have contributed to their lower s-25(OH)D concentrations is strengthened by the fact that most of the other factors known to influence vitamin D concentrations were matched for in the groups or adjusted for in statistical analysis. Factors that affect circulating 25(OH)D concentrations include age, gender, smoking, ethnicity, seasonal effects and dietary intake [11]. The participants in the study sample were almost exclusively of mixed ancestry ethnicity, the two groups were matched for age and gender, while smoking and vitamin D intake were adjusted for in regression analyses. Blood samples for s-25(OH)D determination were obtained across all seasons in both groups, at similar times of the year within the matched pairs. However, it should be noted that since sun exposure was not specifically estimated, it is possible that reduced sun exposure in the AUD group could have contributed to the difference in s-25(OH)D concentrations between the groups. This may require further investigation as Malik *et al*., indicates that reduced sunlight exposure may be the main cause of vitamin D deficiency. The very poor dietary intake of vitamin D in both groups should be noted, but the mentioned limitations in the SAFOODS database regarding vitamin D content of foods [52], limits the interpretive value of these results. The lack of information on dietary supplement use also needs to be considered as a limitation.

Available information on the association between vitamin D and alcohol is limited. Alcoholics have been reported to have reduced circulating vitamin D concentrations [60,61,62], which may be related to the effect of alcohol itself on vitamin D absorption, altered biliary secretion, poor dietary intake or reduced sun exposure [61]. Evidence from work in rodents indicated that chronic alcohol intake may result in reduced serum 1,25(OH)_2_D concentrations due to impaired renal synthesis and/or increased degradation of 1,25(OH)_2_D [63]. More research is clearly needed to elucidate the different mechanisms that contribute to the reduced vitamin D concentrations associated with heavy alcohol intake, specifically in the form of binge drinking.

When compared to available South African information regarding s-25(OH)D concentrations and prevalence of insufficiency/deficiency in children and adolescents, results suggest a higher prevalence of vitamin D insufficiency/deficiency in adolescents in this study sample. A small study in Polokwane (latitude of approximately 24 degrees south) in healthy black children and adolescents found mean s-25(OH)D concentrations to be approximately 50 ng/mL in 6 to 9 year-olds (*n* = 17), 46 ng/mL in 10 to 13 year-olds (*n* = 26) and 36 ng/mL in 14 to18 year-olds (*n* = 15). The same study found that in healthy albino children s-25(OH)D concentrations were approximately 41 ng/mL in 6 to 9 year-olds (*n* = 30), 34 ng/mL in 10 to 13 year-olds (*n* = 36) and 36 ng/mL in 14 to18 year-olds (*n* = 16) [64]. The concentrations in these studies are mostly within the vitamin D sufficiency range (s-25(OH)D concentrations ≥ 30 ng/mL) and much higher than the serum concentrations evident in this study in adolescents of mixed ancestry in Cape Town (latitude of approximately 33 degrees south). In a recent assessment of vitamin D status in a cohort of healthy 10 year-old urban children (*n* = 475) in the greater Johannesburg area (latitude of approximately 26 degrees south), 7% were vitamin D deficient (s-25(OH)D less than 20 ng/mL), while 19% were Vitamin D insufficient (20 to 29 ng/mL) [41]. Seasonal variations in s-25(OH)D concentrations were seen only in white children, with concentrations being significantly higher in white compared to black children during the autumn and summer months [41]. The differences in s-25(OH)D concentrations between this study and the other studies mentioned may be attributable partly to the more southerly latitude of Cape Town. To this effect, a study in Cape Town reported only limited vitamin D synthesis *in vitro* in the winter months from April through to September [65].

Considering the fact that s-25(OH)D concentrations of 30 ng/mL or less are associated with a significant reduction in intestinal calcium absorption [8], the combination thereof with the low calcium intake in this study sample is of concern, especially in the AUD group, who seem to have a greater risk of vitamin D deficiency. The interaction of activated vitamin D (1,25(OH)_2_D) with the vitamin D receptor is needed for the production of calbindin, a calcium-binding protein involved in transcellular calcium transport for intestinal absorption [4], which is the dominant mode of absorption when calcium intake is low or moderate [4].

The low estimated dietary calcium intake of adolescents in this study is in line with previous studies showing poor calcium intake in adolescents [21,66]. Although calcium intake in the AUD group was significantly greater than in the non-AUD group, this difference can be regarded as clinically insignificant since all participants in the sample had intake less than the EARs for calcium for 14 to 18 year-olds (1100 mg). Calcium-rich foods were consumed only eight times per week (about once daily) in both groups, which in all likelihood did not provide the one to two dairy servings per day recommended by the South African Food-Based Dietary Guidelines [67], and likely accounts for the low calcium intake. This is supported by the fact that calcium contribution from milk (first in the ranking of the top five foods/energy containing beverages contributing to calcium intake) per participant per day amounted to only about 100 to 120 mL of milk per day (120 mg of calcium per 100 mL milk) [51]. White bread and crisps (maize- and wheat-based) also featured as major calcium sources, being ranked second and fourth in both groups, respectively. White bread contains 56 mg of calcium per 100 grams and average maize and wheat crisps contain 81 mg of calcium per 100 g [51], however, the bioavailability of calcium from these foods is lower than from dairy foods [5]. 

The combination of inadequate calcium intake and poor vitamin D status seen in this adolescent sample may have negative implications for skeletal health and attainment of peak bone mass, with heavy alcohol use possibly increasing this risk. Firstly, the low calcium intake *per se* in both groups may hamper peak bone mass attainment, as inadequate calcium intake may translate into inadequate calcium absorption and a decrease in peak bone mass in adolescents [68]. Secondly, the vitamin D insufficiency and deficiency on its own may have harmful implications for bone via the effects of hyperparathyroidism and the resultant increased osteoclast activity and bone resorption [69] in these adolescents, with potentially worse impacts in the AUD adolescents due to their increased risk for vitamin D deficiency. Lastly, the poor vitamin D status may compound the effects of the low calcium intake in these adolescents as absorption of calcium at low levels of intake is primarily dependent on the presence of activated vitamin D. 

Depressed bone synthesis has been demonstrated in experimental animal models [70] and alcoholics [71], who have reduced bone density and bone mass, increased fracture susceptibility and increased osteonecrosis [72]. Furthermore, reduced vitamin D concentrations have been observed in alcoholics [60,61,62], which may indirectly interfere with bone metabolism. Acute and chronic alcohol exposure is also known to disturb calcium homeostasis [26,73], partly via effects on vitamin D, but also via alcohol mediated inhibition of intestinal calcium transport independent of vitamin D [73,74]. The notion that alcohol use may negatively impact on bone health in adolescents is supported by the results of a recent prospective study in 109 high school students that examined the association between osteoporosis risk factors and attainment of bone mass over a four year period. The results showed that alcohol had a significant inverse association with bone mineral density while adequate dairy intake, defined as greater than four servings per day, had a significant positive association with bone mineral density [75]. Similarly, Neville and colleagues found a non-significant trend towards an inverse association between alcohol intake and bone mineral density [23]. The negative association between alcohol intake and bone mineral density is further supported by evidence from animal models wherein intermittent binge-like alcohol exposure in adolescent and young adult rats had significant negative effects on bone integrity, including trabecular structure, bone mass and functional strength capacities of bone [27,76,77] and was found to increase osteoclastic resorption [24]. Moreover, a recent laboratory investigation in adolescent rats using binge drinking models demonstrated that binge alcohol exposure can produce disturbances of normal bone gene expression patterns that persist well beyond the phase of active intoxication [78]. Therefore, high levels of alcohol exposure (in binges) may produce both short and long term skeletal damage in the adolescent rat. It can thus be argued that the poor vitamin D status and inadequate calcium intake, exacerbated by the repeated alcohol-induced disruptions in vitamin D and calcium homeostasis in the AUD adolescents, may increase their risk of harmful skeletal outcomes. Furthermore, binge drinking behaviours that begin during late adolescence tend to continue into early adulthood [79] thereby increasing the time that alcohol-related skeletal damage may be occurring in young adults. 

In interpreting the results of this research, the inherent limitations of dietary intake methodology and self-report of alcohol consumption need to be considered. Furthermore, the use of the Chi-square test to assess differences in categorical variables does not allow for adjustment for possible confounders. Thus comparisons done between the two groups using this test are subject to this limitation.

## 5. Conclusions

Bearing in mind the mentioned limitations, it can be concluded from this cross-sectional comparative study that both groups of adolescents had a poor biochemical vitamin D status, with heavy alcohol use in the form of binge drinking, possibly increasing this risk. Furthermore, both groups had a high risk for inadequate calcium intake, which could compound the possible consequences of poor vitamin D status and heavy alcohol intake. It is thus plausible to speculate that the poor calcium and vitamin D status in these adolescents, especially those with AUD, may impact negatively on attainment of peak bone mass and consequently may increase the risk of osteoporosis later in life.

Although the findings in this non-representative sample cannot be directly extrapolated to the adolescent population in the Cape Town area, they provide some indication that vitamin D and calcium status may be less than desirable in this age group and may be compounded by heavy alcohol use, which warrants further investigation. Additionally, the findings of this study require further exploration in longitudinal, well-controlled studies, to confirm the negative associations between heavy adolescent alcohol use and vitamin D status, as well as the impacts of heavy alcohol use on skeletal development and attainment of peak bone mass in adolescents.
